# Effect of geographical location, insect type and cooking method on the nutritional composition of insects consumed in South Africa

**DOI:** 10.3920/JIFF2021.0067

**Published:** 2021-10-05

**Authors:** Z.T. Hlongwane, M. Siwela, R. Slotow, T.C. Munyai

**Affiliations:** 1 School of Life Sciences, University of KwaZulu-Natal, Private Bag X01, Scottsville 3209,South Africa; 2 School of Agricultural, Earth and Environmental Sciences, University of KwaZulu-Natal, Pietermaritzburg, South Africa

**Keywords:** Edible insects, protein, iron, zinc, geographical location, cooking method, amino acids

## Abstract

Edible insects may be a sustainable source of protein and some other nutrients, especially for low-economic-status communities. The current study determined the influence of insect type, geographic location, and cooking method on the nutritional composition of insects. The investigation would contribute to the maximal derivation of the nutritional benefits of insects. Dried samples of four insect types, *G. belina* (mopani worm), *Gynanisa* caterpillar, termite soldiers/workers, and termite alates, were procured from different street vendors across Vhembe district in Limpopo Province, South Africa. *G. belina* samples were cooked by frying, boiling with and without salt addition. Generally, nutrient content varied significantly with insect type and geographic location (p<0.05). Protein content varied from 40 g/100 g in termite alates to 69.75 g/100 g in termite soldiers/workers. Termite soldiers/workers had the highest iron content (range: 545-629.5 mg/kg), whilst *Gynanisa* caterpillar had the highest zinc content (range: 122.14-150.33 mg/kg). Similarly, *Gynanisa* caterpillar had the highest levels of lysine (range: 0.80-4.53 g/100g), threonine (range: 0.79-2.64 g/100g) and isoleucine (range: 0.63-2.33). On the other hand termite soldiers/workers had the highest levels of valine (range: 2.20-3.47 g/100g), leucine (range: 2.49-3.87 g/100g) and phenylalanine (range: 1.38-3.55 g/100g). Cooking method significantly affected nutrient retention. Boiling with salt added resulted in the highest retention of protein and total mineral content (ash), and, therefore, seems a suitable method for cooking insects. The findings indicate that, if optimally selected and cooked, edible insects can contribute significantly to the alleviation of protein, zinc, and iron deficiencies in target communities.

## 1 Introduction

The world’s population is increasing at an alarming rate, and it is projected to reach 9.6 billion by the year 2050 (United Nations Department of Economic and Social Affairs, 2017). The increase in human population will require an increase in food production and yields (Belluco *et al*., 2013). Malnutrition, in its different forms is a global problem, and limited progress is being made to address it. In 2017, it was reported that 821 million people were undernourished in the world; of these, 256 million were found in Africa (Kourimska and Adamkova, 2016). Unfortunately, with the rapid growth in global population, these figures are expected to triple by 2050, provided no efforts are being made to reverse the current situation (van Huis, 2013). The increase in the demand for animal protein causes chicken, beef, fish, and grain prices to increase (Raheem *et al*., 2019). Livestock production is associated with land degradation, water and air pollution, accounting for more than 14% of annual greenhouse gas emissions worldwide (van Huis, 2013; Rojas-Downing *et al.,* 2017). In addition, the growing demand for animal protein will negatively impact the environment, as more greenhouse gases will be emitted, and more land and water will be required (Henchion *et al*., 2017). As a result, climate change will be exacerbated (van Huis and Oonincx, 2017). Rising demand for animal protein will have a negative effect on the environment and food prices. The World Bank (2020) reported that approximately 501 million people in Africa lived below the international poverty datum line of $1.90 a day or less and could not access good quality nutritious food.

Conventional protein sources (meat, fish, and chicken) will not be enough to feed the exponentially growing human population (Kourimska and Adamkova, 2016; van Huis, 2016; World Bank, 2020; Zielinska *et al*., 2015). Therefore, relatively more affordable and sustainable alternative protein sources need to be adopted (van Huis, 2013; van Huis, 2016). Edible insects have been proposed as an alternative protein source, because of their nutritional value, diversity and abundance (van Huis, 2013). In addition, insect farming and breeding can lead to the reductions in the emissions of greenhouse gases (Kourimska and Adamkova, 2016). Adoption of edible insects as an alternative protein source might be a solution that can help alleviate the environmental pressure caused by livestock production (van Huis, 2013). Furthermore, edible insects can reduce malnutrition globally, especially in developing countries where malnutrition, particularly undernutrition, is a major challenge (van Huis, 2013; van Huis, 2016; Zielinska *et al*., 2015).

Edible insects are highly nutritious and considered a healthy food source (Dobermann *et al*, 2017; Kourimska and Adamkova, 2016; Payne *et al*., 2016; van Huis, 2016). They are high in energy, protein, essential amino acids, fat, and minerals, including zinc and iron (Feng *et al*., 2018; Kwiri *et al*., 2014). Thus, edible insects currently play an important role in human nutrition in the developing regions of Africa (Kelemu *et al*., 2015; Manditsera et al., 2018; Niassy *et al*., 2018; Tao and Li, 2018), Asia (Chen *et al*., 2009; Poshadri, 2018), and Latin America (Costa-Neto, 2015; Hurd *et al*., 2019), where they are a common traditional food source. As a result, consumption of edible insects should be encouraged in developed countries and used as a source of food in the future. Jongema (2017), reported that approximately 2111 insect species are consumed around the world of these, approximately 500 species are found and consumed in Africa (Kelemu *et al*., 2015; Manditsera *et al*., 2018; Niassy *et al*., 2018). In Africa, edible insects are consumed for their taste, nutritional value, and as an emergency food source during times of food shortages (Agea *et al*., 2008; DeFoliart, 1997; Mutungi *et al*., 2019). The most widely consumed insects in Africa are Lepidopteran caterpillars, termites, and grasshoppers (Illgner and Nel, 2000; Kachaphulula *et al*.,2018; Kelemu *et al*.,2015; Raheem *et al*.,2019).

Edible insects are subjected to different processing and cooking methods before consumption (Manditsera *et al*., 2019). They are consumed in different forms such as stewed, dried, grilled, roasted, boiled, and as a side dish to a starchy meal, or as a snack (van Huis, 2013). Cooking edible insects changes the colour and texture of edible insects and improves sensory quality through the formation of aromatic compounds (Melgar-Lalanne *et al*., 2019). In addition, cooking of edible insects improves taste, shelf life and safety (Manditsera *et al*., 2019). However, cooking methods might have a negative effect on the nutritional content of edible insects (Lautenschlager *et al*., 2016; Madibela *et al*., 2007). Kinyuru *et al.* (2010) found that toasting and drying grasshoppers resulted in a significant reduction in protein digestibility while no significant change was reported in toasted and dried termites. Manditsera *et al*. (2019) reported that boiling resulted in a decrease in protein content of edible beetles (*Eulepida mashona*) and crickets (*Henicus whellani*) consumed in Zimbabwe. Frying resulted in a decrease in zinc content of mopane worm consumed in Botswana (Madibela *et al*., 2007).

Mopane worms are considered a delicacy and are the most consumed and preferred insect in the Southern Africa (Thomas, 2013; Makhado *et al*., 2014; Baiyegunhi *et al*., 2016). They are processed and cooked using different methods which vary with geographic region (Mutungi *et al*., 2017). In South Africa mopane worms are traditionally consumed dried as a crispy snack, they can also be further boiled, fried or cooked to a tomato stew (Mpucane *et al*., 1996; Illgner and Nel, 2000; Hlongwane *et al*., 2021). Boiling, drying and frying were the most preferred and common traditional methods used to cook mopane worms in South Africa (Hlongwane *et al*., 2021). Traditionally mopane worms are served as a side dish to maize porridge and leafy vegetables (Illgner and Nel, 2000).

Although many insect species are consumed in Africa, the number of species consumed in the different regions, countries, and geographical locations of Africa vary. The latter is due to consumer preferences, socio-economic status, and availability of insect species, which can in turn be affected by natural ecological factors, human activities, such as agriculture and urbanisation, or trade (Anankware *et al*., 2017; Hlongwane *et al*., 2020; Imathiu, 2019; Manditsera *et al*., 2018; Nonaka, 2009). The nutritional composition of insects varies with insect type (genetic factors, species, diet), life cycle stage, environmental factors like geographical location (Klunder *et al*., 2012; Manditsera *et al*., 2019; Mutungi et al., 2019; Nyangena *et al*., 2020), and processing and preparation methods (Kinyuru et al., 2010; Klunder *et al*., 2012; Nyangena *et al*., 2020). While several studies have been conducted to assess the nutritional composition of edible insects consumed in other African countries (Assielou *et al*., 2015; Banjo *et al*., 2006; Christensen *et al*., 2006; Edijala *et al*., 2009; El Hassan *et al*., 2008; Illgner *et al*., 2000; Manditsera *et al*., 2018; Mba and Elekima, 2010; Teffo *et al*., 2007), few have been conducted in South Africa, other than the nutritional composition of *Encosternum delegorguei* Spinola (stink bug) (Teffo *et al*., 2007), and *Hemijana variegate* consumed in Limpopo province (Egan, 2013). The objectives of this study were to determine the: (1) nutritional composition of some major types of edible insects consumed in South Africa, (2) the effect of geographic origin on their nutritional composition, and (3) the effect of cooking method on their nutritional composition. The information will be useful in optimising the use of edible insects to contribute significantly to the alleviation of food and nutrition insecurity prevalent in developing regions, especially sub-Saharan Africa. Data on the potential of edible insects in addressing protein and mineral deficiencies in this region will be particularly useful.

## 2 Materials and Methods

### 2.1 Origin of insects and pre-preparation/cooking of insects

**Figure 1:**
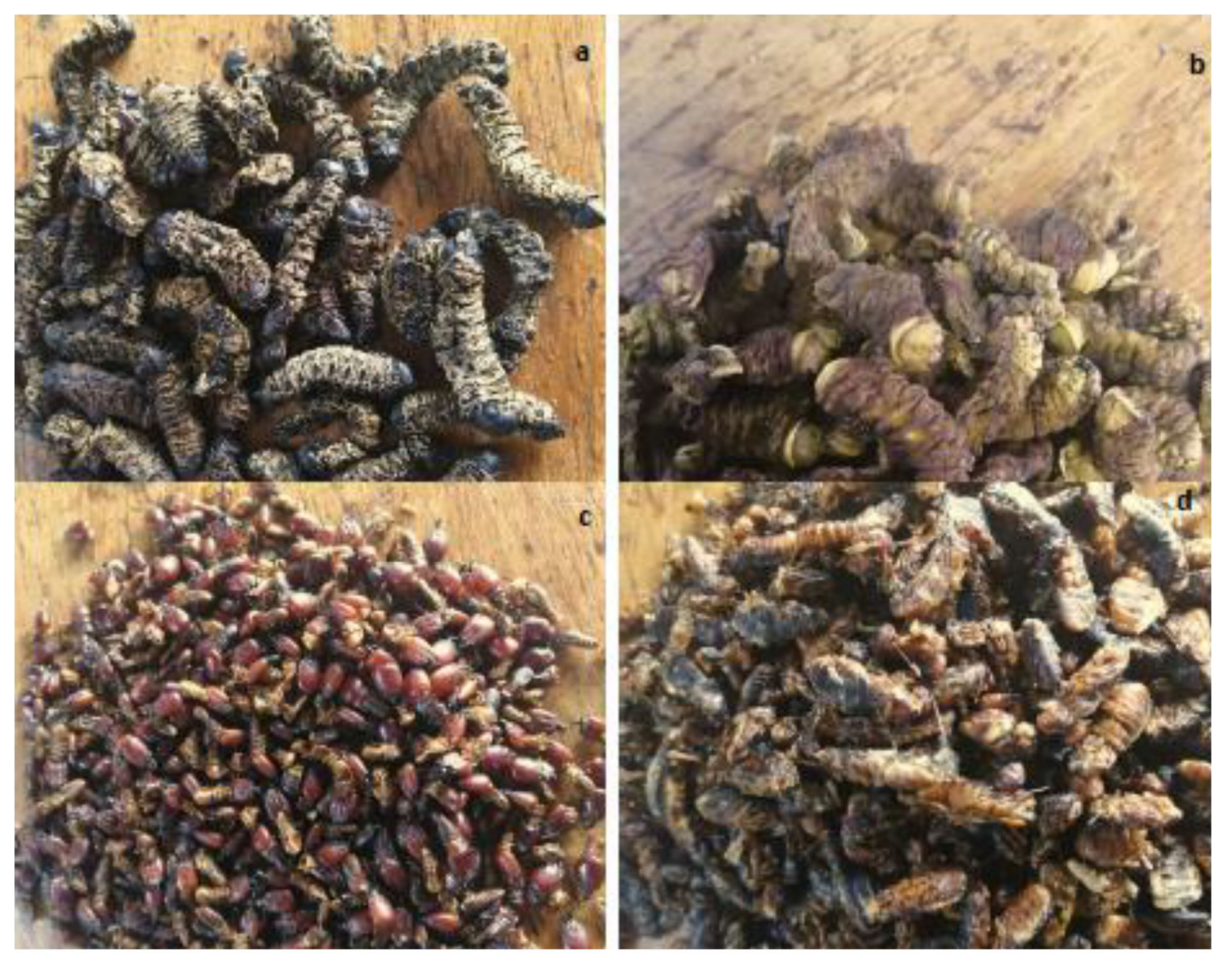
Edible insects included in the study: (a) *Gonimbrasia belina*, (b) *Gynanisa* caterpillar, (c) termite soldiers/ workers, (d) termite aletes

Dried edible insects, i.e *. G. belina* (mopane worm), *Gynanisa* caterpillar, termite soldiers/workers, and termite alates (Figure 1), were purchased from various markets in Vhembe district, Limpopo, in December 2019. The insects were kept in a freezer (≈-18°C) until needed for analysis. Insects bought in Vhembe markets were originally from Zimbabwe, Botswana, Zambia, and around Limpopo Province of South Africa. Traders were asked the geographic origin of the mopane worms they were selling. It was found that the insect traders were from Botswana, South Africa, Zambia and Zimbabwe. The traders sold insects obtained from their respective countries. The traders from South Africa sold insects they had either harvested or bought from middlemen who would have harvested the insects in South Africa. Generally, each trader sold mopane worms obtained from only one geographic location. It was noted that most of the traders were from Botswana.

#### Traditional preparation method of G. belina

Harvesters and traders mentioned that they collected live caterpillars (*G. belina*) from the host tree and then placed them in a bucket. They are then degutted to remove the content of the gut, washed in cold water, then boiled with salt water for 30 minutes, and sundried for one day (Hlongwane *et al*., 2021). Dried *G. belina* are ready for consumption, either dried or further cooked (Egan, 2013).

#### Cooking methods

The effect of cooking method was only investigated on mopani worms (*Gonimbrasia belina*) samples, because of limited resources. Mopane worms were chosen for this experiment as they served as the best example of insects cooked using the methods investigated. Although common methods of cooking and preserving edible insects are often stated in the literature (e.g. Illgner and Nel, 2000; Manditsera *et al*., 2019; Hlongwane *et al*., 2021), *viz*. boiling, frying, salting and drying, the technical details of the methods are not provided. The cooking methods (including technical details) used in the current study were obtained from the study respondents from Vhembe district in Limpopo province of South Africa from where the edible insects were purchased. Dried caterpillars were washed with cold water and subjected to the following cooking treatments: (1) raw (sun dried), (2) boiling in water for 30 minutes, (3) boiling in water with additional salt added for 30 minutes, and (4) fried with oil and salted. Each cooking treatment was performed three times and the mean of the replicate was taken. Recipes and detailed cooking procedures are listed below. The cooking treatments followed traditional cooking methods described by respondents in the Vhembe district, Limpopo (Hlongwane *et al*., 2021). To determine the effect of insect type and geographic location on nutrient content, six replicate samples were analysed, whilst nine replicates were analysed for each cooking method.

#### Scientific laboratory procedures

##### Method A: Recipe for cooking boiled G. belina samples

###### Ingredients

The ingredients used were 5 cups (479 g) of *G. belina,* and 500 ml of water

###### Method

Wash *G. belina* samples in cold water, drain the water and set aside. Put washed *G. belina* samples in a pot and add water. Allow *G. belina* to boil for 30 minutes under medium heat. After 30 minutes remove *G. belina* and let them cool in a container.

##### Method B: Recipe for cooking boiled and salted G. belina samples

###### Ingredients

The recipe consisted of the following ingredients: 5 cups (479 g) of *G. belina;* 500 ml of water, and 5 g (1 teaspoon) of salt

###### Method

Wash *G. belina* samples in cold water, drain the water and set aside. Put washed *G. belina* samples in a pot, add water and salt. Allow *G. belina* to boil for 30 minutes under medium heat. After 30 minutes remove *G. belina* and let them cool in a container.

##### Method C: Recipe for cooking fried G. belina samples

###### Ingredients

The ingredients used were 5 cups (479 g) of *G. belina;* 54 g (4 tablespoon) of cooking oil, 5 g (1 teaspoon) of salt, and 500 ml of water

###### Method

Wash *G. belina* samples in cold water, and soak them in 500 ml of water for 20 minutes. Drain the water and set aside. In a medium pan heat cooking oil and add G. belina. Stir *G. belina* samples occasionally. Allow *G. belina* samples to fry for 20 minutes under medium heat.

###### Average portion size

A sub sample (10 persons) of the study participants (insect traders who were also insect consumers) were asked to dish out (using a cup) the typical amount of the dried insect they would consume in one meal. For simplicity, it was assumed that the typical portion consumed was the same for an adult and child. The mass (size) of each of the ten portion sizes was determined, separately, and the average portion size was calculated. This was done for each insect type included in the study.

### 2.2 Nutritional composition of edible insects

The nutritional composition of edible insect samples was determined using the standard methods of the Association of Official Analytical Chemists (AOAC). Before nutritional analysis, cooked *G. belina* samples were oven dried (60°C) for 24 hours to reduce moisture content. Dried samples were then milled into fine meal with a hammer mill (model SK1, manufactured by Retsch KG, Haan, Germany). Three samples were replicated three times for each cooking method.

To determine protein content of edible insects, samples were measured with a LECO Truspec Nitrogen Analyser (LECO Corporation, St Joseph, Michigan, USA) using the Association of Official Analytical Chemists (AOAC) Official Method 990.03 (AOAC, 2003). Samples were measured in triplicates, and placed into a combustion chamber at 950°C with an autoloader. The following equation (AOAC, 2003) was used to calculate the percentage of protein:

%crudeprotein=%N×6.25.

The total mineral content of the samples was measured using the AOAC Official Method 942.05 (AOAC, 2003). The samples were weighed and placed in a furnace at 550 °C for 24 hours. The minerals remained as a residue of ash in the crucibles after the volatilisation of the organic matter from the samples.

The fat content of the samples was determined following the Soxhlet procedure. The Büchi 810 Soxhlet Fat extractor (Büchi, Flawil, Switzerland) was used for the analysis according to the AOAC Official Method 920.39 (AOAC, 2003).

Amino acids were determined by the hierarchical clustering linear combination (HCLC) method after HCl hydrolysis and derivatization. The method was according to the International Analytical Group (International Analytical Group, 2016) and is briefly described below. The freeze-dried sample was added to a glass vial and 6 N HCl was added. Thereafter, the vial was flushed with argon or nitrogen gas to eliminate oxygen, before the lid was closed. The vial was placed in an oven at 110 °C for 18-24 hours. The vial was removed from the oven and allowed to cool. The hydrolysate was filtered using centrifuge tube filters (Corning® Costar® Spin-X tubes, Sigma-Aldrich, St. Louis, MO, USA). The filtrate was transferred to Eppendorf tubes and allowed to dry using a speedvac, and thereafter reconstituted in borate buffer for derivatization. The borate buffer was transferred into a 200 μl glass insert in a 2 ml glass vial, and 10 μl of either standard solution or diluted sample was added. The 6-aminoquinolyl-N-hydroxysccinimidyl carbamate (AQC) reagent was added, and then the vial was placed in a vortex to ensure that the sample was mixed properly. The vial was then placed in an oven at 55°C for 10 minutes, and then loaded into the autosampler tray for analysis. An H-class Waters Acquity ultra performance liquid chromatography (UPLC) linked to a Waters photodiode array detector (Waters, Milford, MA USA), was used for high-resolution UPLC-UV analysis. The separation was achieved on an Acquity UPLC BEH C18 (2.1 × 150 mm; 1.7 μm particle size) column at 60°C and a flow rate of 0.4 ml/min. Data were collected at a wavelength of 254 nm. An injection volume of 1 μl was used, and gradient separation was performed using solvents A and B from the Waters Accutag kit. The standard dynode, phosphor, and photomultiplier detection system was used.

Gross energy was determined by combusting 1g of the sample in a bomb calorimeter, according to the AOAC Official Method 942.05 (AOAC, 2003). The initial temperature of the calorimeter was recorded (Ti), the sample was ignited, and the final temperature recorded (Tf). The energy value of the sample was computed as: Gross energy (Kcal/g) = (ΔT×Cs)- length of wire burnt (Wt ×1000). Where ΔT = Temperature change (Tf - Ti), Wt = Weight of sample, Cs = Energy equivalent of the bomb system (2464 Cal/g).

Zinc and Iron were analysed using the Agricultural Laboratory Association of Southern Africa (ALASA) Method 6.1.1 (ALASA, 1998). The first step of this process was to freeze-dry the samples in a freeze drier (Edwards, High vacuum international, Sussex, England). Samples were ashed for 24 hours at 550 °C in a furnace. The samples were dissolved in HCl and then HNO3 was added. The samples were analysed using an atomic absorption spectrophotometer. Iron was determined with the Varian SpectrAA atomic absorption spectrophotometer (Varian Australia Pty Ltd, Mulgrave, Victoria), and zinc with the GBC 905AA spectrophotometer (GBC Scientific Equipment Pty Ltd., Dandenong, Victoria, Australia). Only zinc and iron were included in the results. Other minerals (phosphorus, calcium, sodium, magnesium, potassium, manganese and copper) were excluded from the results because the focus was only on the problematic minerals i.e. zinc and iron, which are generally deficient in the diets of a large proportion of populations in the Sub-Saharan African countries, including South Africa.

Estimated Average Requirements (EARs) is one of the four Dietary Reference Intakes (DRIs). The EAR is the recommended DRI for assessing the nutritional status of population groups, defined by demographic profiles, including age, gender and lifecycle stage (Institute of Medicine, 2006). The EAR is the amount of a nutrient that is estimated to meet the needs of 50% of people in a defined population group and can be used to estimate the prevalence of inadequate nutrient intakes (EAR cut-point method The EAR cut-point method can be used for most nutrients, but cannot be used for energy because energy intake and expenditure are highly related) (Murphy *et al*., 2006; Institute of Medicine 2001).

In the current study, the formula below was used to calculate the % contribution of insect meal portions to the EARs of the different age categories:

%EARmet=Proteincontentinusualinsectmealportionsize/(EAR)×100

The usual insect meal portion size was determined as described in section 2.1. The same procedure was used to determine the % contribution to EARs for the other nutrients, i.e. zinc and iron.

### 2.3 Statistical analysis

One Way ANOVA was performed to determine if insect type, geographic location and cooking methods had an effect on the nutrient content of the edible insects. If there was significant effect, the Tukey HSD test was performed to separate means. The analysis was performed using IBM SPPS version 25 software (SPSS Inc. Chicago, IL, USA).

## 3 Results and discussion

### 3.1 Nutritional composition of the insects

#### 3.1.1 Proximate composition

Table 1 shows proximate nutritional composition of edible insects collected from different countries. Overall, *G. belina* samples from South Africa, had the highest levels of protein, fat, and energy compared with *G. belina* samples from other countries (Table 1).

One way ANOVA showed that there were significant differences (p<0.05) in the nutrient content of different insect types found in the same geographic location. For example, in SA, the four types of insect studied had different levels of protein and ash (total mineral content) and similar trends were observed in Botswana and Zimbabwe (Table 1). The current results are similar to the findings of several previous studies (Christensen *et al*., 2006; Kim *et al*., 2016; Kuntadi *et al*., 2018; Mba and Elekima, 2010; Musundire *et al*., 2016; Siulapwa *et al*., 2012). Siulapwa *et al.* (2012) investigated the nutritional composition of edible insects consumed in Ndola district, Zambia. The insects studied included *G. belina* (mopane worm), *Gynanisa maja*, *Ruspolia differens* (katydid), and *Macrotermes falciger* (termite), and found that *G. belina* contained higher protein content, whereas *Gynanisa maja* contained higher mineral content compared with other species. Similarly, Musundire *et al.* (2016) investigated the nutritional composition of fourteen edible insects consumed in Zimbabwe, and found that *G. belina* contained the highest protein content compared with other species. However, Kim *et al*. (2019) studied the proximate composition and mineral content of five edible insect types consumed in Chungnam, South Korea, and found that there were variations in the nutritional composition of the insects, with *Oxya chinensis* containing the highest protein content. In contrast, *Verlarifictorus aspersus* contained the highest zinc and iron content compared with other species. It is noted that variations in the nutritional composition of different insect types from the same geographic location may be caused by diet, genetic differences, sex, and life cycle stages (Imathiu, 2019; Kim *et al*., 2019). Different insects feed on different materials, which may vary in nutritional composition. Liu *et al*. (2017) reported variations in nutritional composition of the black soldier fly during different phases of the life cycle. A higher nutrient content was reported in the larvae stage compared to the adult stage (Liu *et al*., 2017). These results suggest that insect types can be selected and recommended for consumption based on their high nutrient content. In the current study, termite soldiers/workers from South Africa would be recommended for consumption compared to other insects, because they were higher in protein relative to the other insect types.

**Figure 2:**
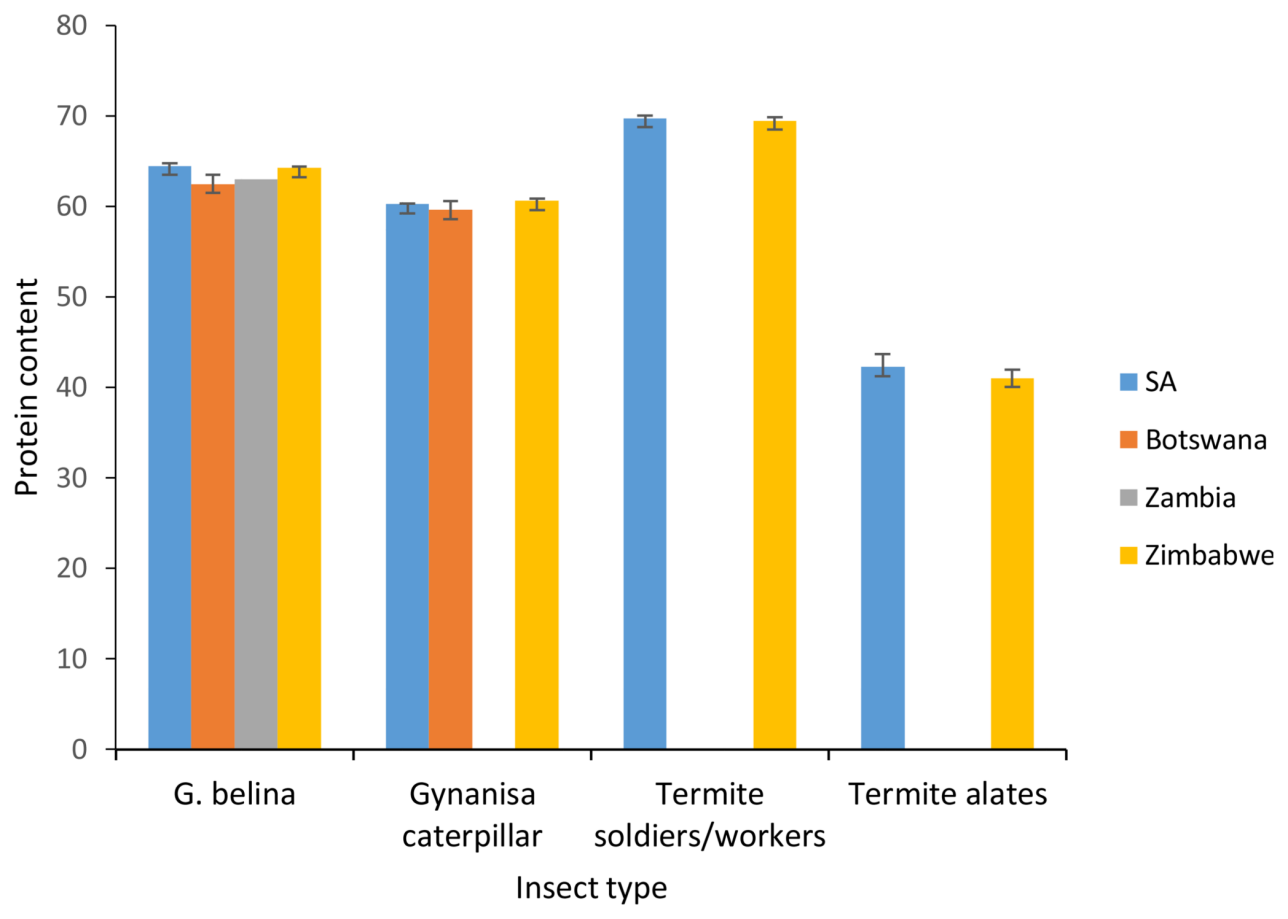
Effect of insect type and geographical location on the protein content of edible insects

Furthermore, one-way ANOVA indicated that there were significant differences in the proximate composition of edible insects across the four countries (Zimbabwe, Zambia, Botswana and South Africa) included in the current study (Table 1). This is the first study comparing the proximate composition of the same insect types found in these countries. The protein content of *G. belina* samples collected from SA was significantly higher (64.47 g/100 g) than the protein content of the samples collected from the other countries, i.e. Zimbabwe 64.26 g/100 g, Botswana 62.46 g/100 g and Zimbabwe 62.99 g/100 g). On the other hand, the protein content of *Gynanisa* caterpillar samples collected from Zimbabwe was significantly higher (60.61 g/100 g) than that of *Gynanisa* caterpillar samples collected from the other countries of the current study. Samples of termite soldiers/workers from SA had significantly higher protein content (69.75 g/100 g) compared with termite soldiers/workers samples from Zimbabwe. Similarly, the protein content of termite alates samples from SA was significantly higher (42.25 g/100 g) than that of termite alates (41.03 g/100 g) samples from Zimbabwe. The trend of protein content of the edible insects is further depicted in Figure 2.

The protein content of *G. belina* samples from SA reported in the present study (64.47 g/100g) is similar to the protein content that was reported by Dreyer (1982), (62 g/100 g) for *G. belina* samples from the same country (SA). However, the protein content (41.03- 64.26 g/100 g) of Zimbabwe insect samples reported in this study is within the range of the protein content (54 -58 g/100 g) of Zimbabwe insects reported by Dube *et al.* (2013). On the other hand, the protein content of *G. belina* samples from Botswana (62.46 g/100 g) reported in this study is higher than the protein content (60.4 g/100 g) of *G. belina* from Botswana reported by Madibela *et al.* (2007). Similarly, the protein content (62.99 g/100 g) of *G. belina* samples from Zambia reported in the present study is higher than the protein content of *G. belina* samples from Zambia reported by Siulapwa *et al.* (2012).

The differences in the proximate composition of edible insects from different countries may be partly attributed to differences in the processing methods (including drying) used. The different processing methods can have different effects on nutrient retention as described and discussed later (Section 3.2). Environmental factors like climate, and soil and vegetation type likely contributed significantly to the observed differences in the nutritional composition of the same insects obtained from different countries (geographic location). Most insects utilise different types of vegetation as feed, and the differences in the chemical composition of the vegetation types would result in differences in the nutritional composition of the insects. For example, there were significant differences in the protein content between *G. belina* sourced in South Africa and Zimbabwe likely due to differences in the chemical composition of feed utilised by the insects in the different countries. The feed of this insect type could be the same in the two countries, but different in chemical composition, due to differences in environmental factors (for example soil type) prevalent in the different specific ecological locations. However, there was no significant difference in protein content between *G. belina* sourced in Zambia and Botswana, which seems to contradict the explanation provided directly above. As stated earlier, despite being in different countries (geographic locations), the same insect type (*G. Belina)* likely fed on the same tree species. There is also a possibility that the chemical composition of the same tree species located in the different geographic locations (Zambia and Botswana) was similar. Although there may be local differences in minerals in the soils in which the tree grows, in some cases there may not be substantial enough differences to translate to detectable differences in protein as measured in the insects. Some insect types, e.g. termites, derive nutrients from the soil. Therefore, differences in soil chemical composition across different geographical locations may affect the nutrition composition of insects, especially their mineral composition. These results suggest that, to ensure a higher protein intake, termite alates and *G. belina* samples from South Africa would be recommended for consumption, because they contained a higher protein and energy content compared with other insect types. As will be discussed later (sub-section 3.1.2), in addition to having a high protein concentration, samples of these insect types sourced in South Africa had a good amino acid profile, including appreciable concentrations of lysine (lysine is deficient in cereal grains, the predominant staples in sub-Saharan African countries). It is noted that termite soldiers had a low fat content compared to the other insect type. The low fat content was compensated by a high protein content (Table 1), which would be of nutritional advantage in addressing protein deficiency, which is prevalent among the majority of population groups in sub-Saharan Africa.

##### Estimated Average Requirement (EAR) met for protein

The ranges of the percentage contribution of the insects studied relative to the Estimated Average Requirement (EAR) met for protein amongst different age groups (Table 2), indicated that the insects would contribute significantly to addressing protein deficiencies, 89.5-160.4% for 4-8 years and 29.9-53.6% for childbearing women (19-50) years (Hlongwane *et al*., 2020). Protein deficiency is a major health problem in developing countries, particularly Southern Africa (Muller and Krawinkel, 2005). Diets of most people in Southern Africa are generally deficient in protein, zinc and iron leading to deficiencies in these nutrients and poor health outcomes. Therefore, *G. belina* consumption would have a huge positive health impact in Southern Africa, especially the low economic status rural communities who are highly vulnerable to malnutrition, including protein deficiency (Govender *et al*., 2017; Modjadji and Madiba, 2019; Voster, 2010). *G. belina* have quite a high protein content (62.99-64.47 g/100 g), and are consumed in relatively higher portion sizes than the other insects. Therefore, they would be more effective in addressing protein deficiency than the other insects, including termite spp. (termite soldiers/workers and alates). Despite having a higher protein content than *G. belina*, they are consumed in smaller portion sizes (Table 2).

#### 3.1.2 Amino acids

According to one way ANOVA, the essential amino acid content of edible insects collected from different countries (Table 3) showed significant differences. The results show that *Gynanisa* caterpillar obtained from Zimbabwe had the highest lysine content followed by the different termite types obtained from Zimbabwe and SA, whereas *G. belina* samples obtained from all the three countries had the lowest lysine content. The different types of termites sourced in SA and Zimbabwe had higher levels of methionine relative to *Gynanisa* caterpillar and *G.belina*, which generally had similar levels of methionine. *Gynanisa* caterpillar and termite workers/soldiers sourced in Zimbabwe had the highest total essential amino acids content followed by termite alates from SA and termite alates from Zimbabwe whilst *G. belina* samples from all the four countries had the lowest total essential amino acid content. *Gynanisa* caterpillar from Zimbabwe had the highest total essential amino acid content, it also had the highest histidine and threonine contents.

Siulapwa *et al.* (2012), studied essential amino acids of four edible insect species in Zambia, and found that amino acid content varied significantly among species, and insects contained a considerable amount of amino acids. The influence of geographical location on the amino acid profile of insects observed in this study can be attributed to environmental factors and differences in processing methods (including drying and cooking methods) used in the different geographical regions as discussed earlier. For example, differences in the mineral composition of soils from different geographical region would affect the amino acid profile and minerals. High sulphur soil content will result in high concentrations of sulphur containing amino acids, such as methionine and cysteine. Similarly, the amino acid composition of insects is dependent on the chemical composition of vegetation consumed.

Cereal and legume grains are leading food sources in the diets of the majority of populations in most of the sub-regions of Sub-Saharan Africa, including southern Africa, yet, they are generally deficient in lysine and tryptophan and methionine, respectively (van Huis *et al*., 2013). Cereal-legume composite foods are usually used so that the two grain types (cereals and legumes) complement each other with regard to amino acid profile. However, legumes are not as available and accessible as cereal grains, as a result, monotonous cereal-based diets are popular. From the current results, it is clear that edible insects contain a considerable amount of amino acids, including lysine and tryptophan and methionine. Therefore, supplementing the diets of the populations mentioned above with edible insects would significantly address deficiencies in essential amino acids, such as lysine and methionine, as similarly suggested by Ebenebe *et al*. (2017) and Igwe *et al*. (2012). The insect types can be ranked in nutritional superiority with respect to total essential amino acid content as *Gynanisa* caterpillar from Zimbabwe> termite soldiers/worker from Zimbabwe > termite alates from SA> termite alates from Zimbabwe> *G. belina. Gynanisa* caterpillar and termite soldiers/workers from Zimbabwe should be prioritised in human diets for higher lysine and methionine content intake. However, climate and land use changes are a major threat, and result in the decline in the abundance and diversity of edible insects in South Africa (Hlongwane *et al*.,2021; Teffo *et al*.,2007). As a result, the decline in the availability of insects would affect the well-being of the communities who depend on them for food and nutrition security. Therefore, edible insects could potentially be farmed to increase their availability and accessibility.

#### 3.1.3 Mineral element composition, focusing on zinc and iron

Table 4 shows mineral element composition of insects from the four countries included in the current study. The zinc content of *G. belina* samples (150.33 mg/kg) from South Africa reported in this study is similar to zinc content (140 mg/kg) reported by Dreyer and Wehmeyer (1982) for *G. belina* samples obtained from the same country. Similarly, the zinc content of *G. belina* samples (108.3 mg/kg) from Zimbabwe reported in this study was lower than zinc content (142 mg/kg) reported by Glew *et al*. (1999) for *G. belina* samples from the same country. On the other hand, zinc content of *G. belina* samples (133.3 mg/kg) from Zambia reported in this study was lower than the zinc content of *G. belina* samples (260.7 mg/kg) reported by Siulapwa *et al.* (2012) in the same country. Differences in zinc content of *G. belina* samples from Zambia could be due to a difference in the processing methods used to prepare samples before analysis *G. belina* samples were degutted and boiled for 1 hour and smoked on a grill above the fire or directly deposited in hot coal for one day (Cloutier, 2015). Boiling mopane worm samples for longer might result in minerals leaching in a cooking medium (Manditsera *et al*., 2019). All the insect types studied also contained appreciable levels of other mineral elements, sodium, phosphorus, calcium, magnesium, potassium, copper and manganese (Supplement Table 5). The current report seems the first on the mineral composition of termites and *Gynanisa* caterpillar from Zimbabwe, South Africa, and Botswana. However, termites and caterpillars consumed in Africa have been found to contain high zinc levels. For example, Adepoju and Ajayi (2016), Kinyuru *et al.* (2013), Madibela *et al.* (2007), Mbah and Elekima (2010), Siulapwa *et al.* (2012) reported that zinc content of termite and caterpillar ranged from 8-16.9 mg/100 g and 8-14 mg/100 g respectively.

One way ANOVA and the results in Table 4 show that there are significant differences in the mineral element composition of different insect types found in the same geographic region. For example, in South Africa the four types of insects studied, i.e. *G. belina, Gynanisa* caterpillars, termite soldiers/workers, and termite alates, had different levels of minerals including zinc and iron. Similar trends were observed for the insects obtained from Zimbabwe and Botswana. Similar results were reported in several previous studies (Kim *et al*., 2016; Kuntadi *et al*., 2018; Payne *et al*., 2015; Siulapwa *et al*., 2012). Payne *et al*., (2015) conducted a study of mineral composition of five edible insects consumed in Zimbabwe (*G. belina*, *Gynanisa maia*, *Macrotermes* spp., *Cirina forda*, and *Encosternum delegorguei*), and found that *G. belina* contained the highest iron content, whereas *Encosternum delegorguei* had the highest zinc content. On the other hand, Akullo *et al*. (2018) investigated the nutritional composition of three insects commonly consumed in Lango region, northern Uganda, and found that *Macrotermes bellicosus* (termite) contained the highest iron content, whereas the African cricket *Brachytrupes spp.* contained the highest zinc content. As discussed earlier for proximate composition, including total mineral content (ash) (Section 3.1), the factors that likely contributed to differences in the mineral element content of different insect types found in the same geographic region are diet, genetic factors, life cycle states, specific growth environment (niche), and sex (Imathiu, 2019; Kim *et al*., 2019).

One way ANOVA and the results in Table 4 also indicate a variation in the mineral composition of the same insect type across the countries included in the study. The zinc content of *G. belina* samples collected from South Africa was significantly higher than that of the *G. belina* samples collected from the other countries included in the study. Similarly, the zinc content of *Gynanisa* caterpillar samples collected from South Africa was significantly higher than that of the same insect collected from other countries. Termite soldiers/workers samples from South Africa (142.66 mg/kg) had significantly higher zinc content than termite soldiers/workers samples from Zimbabwe (131.16 mg/kg). On the other hand, there were no significant differences (p>0.05) in zinc content of termite alates samples collected from South Africa and Zimbabwe. The iron content of *G. belina* samples collected from Zimbabwe was significantly (p<0.05) higher than that of *G. belina* samples collected from other countries. However, the iron content of *Gynanisa* caterpillar samples from South Africa was significantly higher than that of *Gynanisa c*aterpillar samples from other countries. Termite soldiers/workers samples from Zimbabwe (629.5 mg/kg) had significantly higher iron content compared with termite soldiers/workers samples from South Africa (545 mg/kg). Similarly, the iron content of termite alates samples collected from South Africa (307.67 mg/kg) was significantly (p>0.05) higher than that of termite alates samples from Zimbabwe (177.17 mg/kg). As was discussed for the other nutrients (Section 3.1) environmental factors such as climate, soil and vegetation type likely were significant contributors to the differences in the mineral element content of the same insects obtained from different countries (geographic location). In addition, as already discussed, differences in the processing methods used to process the same insect types in the different countries probably contributed to the varied mineral element content of the same insect type obtained from the four countries considered.

The iron and zinc contents of the insects of this study agree with the trends stated in the literature, which show that edible insects are high in several mineral elements, including zinc and iron. In fact, the literature indicates that, generally, insects are higher in iron compared to chicken and beef, the popular, but expensive protein sources (Hlongwane *et al*., 2020; Rumpold and Schluter, 2013). Zinc and iron deficiencies are a common health problem in many low income countries (Siwela *et al*., 2020). Zinc and iron are deficient in the diets of many South Africans (Motadi *et al*., 2015). In addition, diets are made up of mainly carbohydrate-rich food, with a low intake of animal protein, dairy products, and fruits, which contribute less iron, protein, and zinc intake (Mamabolo *et al*., 2006; Motadi *et al*., 20150). Therefore, edible insects should be recommended for intake to combat iron and zinc deficiencies, which are prevalent and of health concern in South Africa, as stated earlier. Furthermore, the current study results suggest that, when insects are used to address iron and zinc deficiencies in specific geographic regions (e.g. in different countries), specific insect types that are high in the mineral element of interest should be determined for the specific country. On the basis of the results of the current study (Table 4), termite soldiers/workers should be recommended for consumption in Zimbabwe and *Gynanisa* caterpillar in SA, should be recommended. Furthermore, the trends of variation of mineral element content of the same insect across geographical location (countries in the current study) suggest that geographic regions can be mapped for obtaining maximal levels of target mineral elements (zinc and iron in the current study) specific insect types.

##### Estimated Average Requirement (EAR) met for iron and zinc

All insect types from different countries would contribute a significantly high percentage of EAR (107.7-381%) for iron for 4-8 years old children, but would have a lower percentage contribution to percentage EAR (30-71%) for iron for the groups of child-bearing women ages (19-30) and (31-50) (Christensen *et al*., 2006; Mwangi *et al*., 2018). Child bearing women have a high demand for dietary iron because of loss of this mineral through menstruation. Therefore, insects would not be an effective food source to address iron deficiencies among the latter two population groups. All the insect types from different countries similarly would contribute a high percentage of EAR for zinc for 4-8 years old children (108- 152%), except for termite soldiers/workers, which would contribute a much lower percentage of EAR (50.3-54.5%). The usual portion size of the termite soldiers/workers was much lower in weight than the weight of the usual portion size of other insect types, hence it contributed much less zinc intake. For the same reason as iron, child bearing women have a high demand for other minerals including zinc. Therefore, the usual portion of different insect types would contribute a much lower percentage for zinc (Table 5).

Protein and mineral deficiency lead to several health conditions which are common in developing countries. Mineral deficiency results in the high proportion of unhealthy, morbid and less productive people among the populations in these regions (Govender *et al*., 2017; Siwela *et al*., 2020). The affected developing countries need alternative sustainable and economically accessible food sources to address the health problems from challenges caused by nutrient deficiencies. The current study results clearly show that insects would be suitable alternative food sources to address protein and mineral deficiencies in developing regions, and, thereby, improve the health and well-being of the population.

#### 3.2 Effect of cooking methods on the nutritional composition of insects

**Figure 3:**
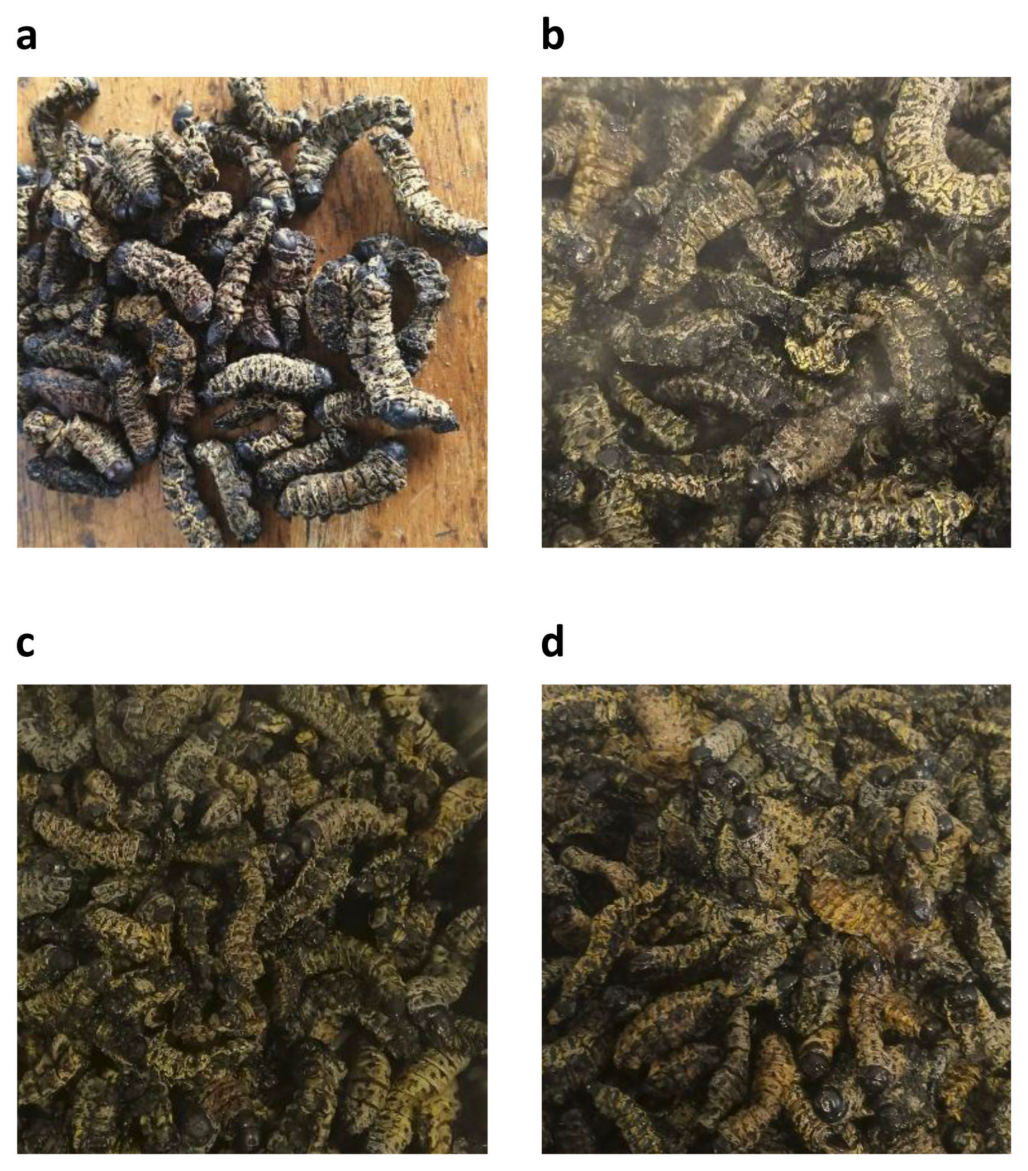
Pictures representing representing uncooked dried *Gonimbrasia belina* (control), and *Gonimbrasia belina* samples cooked using different methods (a) dried (uncooked), (b) boiled, (c) boiled and salted and (d) fried

Figure 3 is comprised of pictures representing uncooked, dried *G. belina* (control), and *G. belina* samples cooked using different methods. The *G. belina* samples that were boiled in water alone (Figure 3 (b)) appear as light as the control, but are swollen, obviously due to water absorbed during boiling. In contrast, samples of *G. belina* samples that were boiled in water with salt added (Figure 3 (c)) and the fried samples (Figure 3 (d)) look darker, but almost the same size and shape as the control, which is likely because they absorbed little water during cooking. The mopane worm samples boiled with salt addition were darker because they absorbed less water than the samples to which salt was not added. The darker samples maybe less acceptable to consumers with respect to appearance as well as texture, which tends to be firmer. However, the flavour of these maybe improved by salt addition. In Southern Africa dried *G. belina* are further cooked using different cooking methods (frying, boiling, stew, roasted) so that they can be consumed as a side dish to a starchy staple e.g. maize meal stiff porridge (pap) (van Huis, 2013), a very popular staple food in Southern Africa. On the other hand, dried *G. belina* are consumed as a snack.

##### 3.2.1 Effect of cooking methods on proximate composition

Results from One way ANOVA show that cooking method had a significant effect on the nutrient content of the insects. Table 6 shows the effect of different cooking methods on the proximate composition of *G. belina* samples obtained from South Africa. For all three samples of *G. belina* boiling, resulted in a significant increase (p <0.001) in their protein content compared with the controls (dried *G. belina* samples). Similarly, boiling with addition of salt resulted in a significant increase (p< 0.001) in the protein content of *G. belina*. On the other hand, frying resulted in a significant decrease in the protein content of the *G. belina* samples compared with the controls. Some researchers (Medigo *et al*., 2018; Nyangena *et al*., 2020) found that boiling resulted in a significant increase in protein content of *Tenebrio molitor, Ruspolia deifferens, Spodoptera littoralis* and *Acheta domesticus*. On the other hand, frying resulted in a significant increase in protein content of edible insects (Medigo *et al*., 2018; Nyangena *et al*., 2020). Other researchers found that boiling with or without salt as well as frying resulted in a significant decrease in protein content of *G. belina, Eulepida mashona, Hemijana variegata* and *Henicus whellani* (Egan, 2013; Madibela *et al*., 2007; Manditsera *et al*., 2019). Differences in protein content as a result of cooking can be ascribed to differences in boiling and frying time. For example, in the current study, insects were boiled for 30 minutes and fried for 15 minutes, whilst in other studies the insects were boiled for an hour (Egan, 2013; Madibela *et al*., 2007). An increase in protein content during boiling in water alone or with salt added in this study could be attributed to the observed decrease in other nutrients during cooking. Therefore, there is a proportionate increase in protein. In a similar study, Nyangena *et al*. (2020) gave the same reason for an increase in protein content due to the observed decrease in fat content. On the other hand, the decrease in protein content during frying could be attributed to the protein leaching out of the insect sample into the cooking medium. These results suggest that, to increase protein intake per unit weight of cooked insects, boiling should be recommended. Generally, frying increased energy content of *Gonimbrasia belina* samples, whilst, boiling resulted in a decrease in the energy content of the insect samples. During frying the samples absorbed fat, increasing energy level. The decrease in energy content during boiling can be attributed to loss of fat (Table 6).

##### 3.2.2 Effect of cooking method on amino acid profile

Table 7 shows the effect of different cooking methods on essential amino acids of *G. belina* samples obtained from South Africa. Overall, the results show that boiling in water alone or in water with salt added decreased most essential amino acids (histidine, threonine, lysine and valine) compared with frying. However, boiling in water alone and boiling in salt resulted in a significant increase in methionine content. On the other hand, frying resulted in decrease in threonine, lysine, and valine content, but resulted in an increase in histidine and methionine content.

Previous studies have shown that different cooking methods such as boiling with or without salt added, and frying resulted in a significant decrease or increase in amino acid content in meat and fish (El-Lahamy *et al*., 2019; Erkan *et al*., 2010; Oluwaniyi *et al*., 2010; Zhang *et al*., 2014). For example, Zhang *et al.* (2014) found that boiling rabbit meat resulted in a significant increase in all essential amino acids whilst, frying resulted in a significant decrease in lysine, threonine, isoleucine and valine content. A decrease in some amino acids mentioned above can be attributed to several chemical reactions occurring during cooking, such as transamination, and the Maillard reaction, deamination, racemisation, hydrolysis, and desulfuration (Medigo *et al*., 2018; Nyangena *et al*., 2020). The current study indicate that frying or boiling with or without salt would reduce the potential of insects for addressing deficiencies of some essential amino acids in the diets of the populations that are vulnerable to nutrient deficiencies. It is particularly of concern that both frying in fat and boiling in water with or without salt added result in a decrease in the lysine content of the insect samples. In sub-Saharan Africa, diets based on cereal grains are generally the most popular, yet, cereal grains are deficient in lysine. Several studies have shown that supplementing food products with insect flour increase nutritional composition (e.g. Ayensu *et al*., 2019; Kowalczewski et al., 2021; Zielinska and Pankiewicz, 2020). For example, Awobusuyi *et al.* (2020) found that supplementing wheat biscuits with termite flour resulted in a significant increase in lysine content and other essential amino acids. Therefore, to increase lysine intake, dried insects should be ground and incorporated as an ingredient in food products such as bread, biscuits, and cereal grain porridges.

Overall, boiling without or with salt added and frying resulted in a significant increase in ash content of all samples of *G. belina* compared with the corresponding controls. Similar results were reported previously (Dreyer and Wehmeyer, 1982; Edijala *et al*., 2009; Klunder *et al*., 2012; Nonaka, 2009). An increase in the ash content during boiling was likely due to the concentration effect proportionate to the decreasing concentration of the other nutrients. For example, fat content decreased in boiled *G. belina* samples, whilst their ash content increased. The same reason, i.e. concentration effect, for an increase in ash content was suggested by Nyangena *et al*. (2020) In addition, for the samples where salt was added, an increase in ash content was partly due to the fact that salt is a mineral. These results suggest that to maximise mineral intake from the consumption of insects, they should be boiled with an addition of salt.

##### 3.2.3 Effect of cooking methods on mineral element content of insects, focusing on iron and zinc

Table 8 shows the effect of different cooking techniques on the mineral composition of *G. belina* samples obtained from South Africa. Generally, boiling with or without salt and frying resulted in a significant increase in iron and zinc content of *G. belina* samples. Results reported by other authors (Madibela *et al*., 2007; Manditsera *et al*., 2019), found that boiling and frying result in an increase or decrease of mineral content of edible insects. For example, Manditsera *et al*. (2019) investigated the effect of domestic cooking on protein and mineral content of wild harvested edible insects, and found that boiling and frying edible insects resulted in an increase in iron content, however boiling and frying resulted in a decrease in zinc content of edible insect. Similarly, Madibela *et al.* (2007) found that frying resulted in a significant decrease in zinc content of *G. belina* consumed in Botswana. However, El-Lahamy *et al*. (2019) reported that boiling and frying did not affect zinc and iron content of Sudanese tree locust. There were no reports of previous studies on the effect of boiling with an addition of salt in mineral content of edible insects. The increase in zinc and iron during frying and boiling without salt corresponds to an increase in ash content, the reasons for an increase in ash were explained earlier. Salt is a mineral; therefore, addition of salt will result in an increase in total mineral content of *G. belina* samples. These results suggest that, to maximize intake of zinc and iron per unit weight of *G. belina* samples boiling and frying would be the recommended cooking methods.

## 4 Conclusions

The nutritional composition of the edible insects studied varied with insect type. Samples of the termite soldiers/workers were found to be the most nutritious in protein and iron content, whilst *Gynanisa* caterpillar had the highest zinc content. Therefore, edible insects have a potential of alleviating protein, zinc and iron deficiencies around the world. In addition, edible insects’ consumption contributes to food and nutrition security in developing countries. This is directly linked to United Nations SDG goal 2: End hunger, achieve food and nutrition security and promote sustainable agriculture. Thus, consumption of insects should be broadened beyond the traditional population groups. Alternatively, it would be beneficial to recommend the consumption of different types of insects to leverage their different advantages in terms of concentration of specific nutrients. The study further demonstrated that the nutritional composition of the same insect type can vary with geographical location, which suggests the need to identify and map geographic areas that are superior sources of highly nutritive specific insect types.

Cooking methods were found to have different effects on the concentrations of different nutrients in *G. belina* samples. Overall, the results indicate that boiling would be the most recommended method for cooking insects as it retained the highest levels of protein and a significant percentage of total mineral content (ash). Our results showed that insects are a good source of protein, zinc and iron.

## Figures and Tables

**Table 1:** Proximate composition and energy content of edible insects harvested from different localities ^1^

Treatments	Moisture	Protein	Fat	Ash	Gross energy (MJ/kg)
*G. belina* - SA	7.11^f^±0.26	64.47^g^±0.31	14.49^d^±0.82	11.75^d^±0.46	20.4^b^±0.17
*G. belina* - Botswana	6.43^d^±0.13	62.46^f^±0.10	12.11^b^±0.53	15.68^f^±0.12	19.11^e^±0.23
*G. belina* - Zambia	6.93^e^±0.10	62.99^f^±0.28	12.48^c^±0.41	13.64^e^±0.14	19.57^d^±0.22
*G. belina* - Zimbabwe	6.86^e^±0.15	64.26^h^±0.18	12.49^c^±0.10	11.83^d^±0.16	19.86^c^±0.19
*Gynanisa* caterpillar – SA	6.36^d^±0.08	60.26^d^±0.09	11.62^b^±1.28	16.41^g^±0.35	19.32^e^±0.10
*Gynanisa* caterpillar – Botswana	5.72^b^±0.18	59.60^c^±0.53	11.77^b^±0.49	15.62^g^±0.47	19.59^d^±0.05
*Gynanisa* caterpillar - Zimbabwe	6.10^c^±0.19	60.61^e^±0.27	12.11^c^±0.22	14.84^e^±0.43	19.61^d^±0.15
Termite soldiers/workers - SA	6.94^e^±0.15	69.75^i^±0.30	7.97^a^±0.35	7.50^c^±0.14	20.28^b^±0.19
Termite soldiers/workers - Zimbabwe	6.89^e^±0.20	69.48^j^±0.35	7.49^a^±0.19	7.97^c^±0.10	20.41^b^±0.05
Termite alates - SA	3.88^a^±0.94	42.25^b^±1.04	49.5^e^±1.75	5.53^b^±0.51	28.08^a^±0.22
Termite alates - Zimbabwe	7.07^f^±0.41	41.03^a^±0.95	51.91^f^±0.74	4.34^a^±0.35	28.57^f^±0.26
p values	**0.001**	**0.001**	**0.001**	**0.001**	**0.001**

1 Mean±SD, mean of six replicates; Means marked by different letters in the same column are significantly different, (p<0.05). SA=South Africa. Nutrient values are on a dry matter basis (g/100 g).

**Table 2:** Percentage of the Estimated Average Requirement met for protein for different age groups from the consumption of usual portions of dried edible insects from different countries

4-8 years old children					
Insect samples	Protein g/100 g	Average portion size (g)	Protein in meal portion(g)	EAR (g/day)	% of EAR met
*G. belina* - SA	64.47	45.39	29.26	18.24	160.4
*G. belina* - Zimbabwe	64.26	45.39	29.17	18.24	159.9
*G. belina* - Zambia	62.99	45.39	28.56	18.24	156.7
*G. belina* - Botswana	62.49	45.39	28.36	18.24	155.5
*Gynanisa* caterpillar - SA	60.37	40.49	24.44	18.24	134.0
*Gynanisa* caterpillar - Zimbabwe	60.61	40.49	24.54	18.24	134.5
Gynanisa caterpillar - Botswana	59.61	40.49	24.14	18.24	132.3
Termite soldiers/workers - SA	69.75	15.27	10.65	18.24	58.4
Termite soldiers/workers - Zimbabwe	69.48	15.27	10.61	18.24	58.2
Termite alates - SA	42.27	39.77	16.81	18.24	92.2
Termite alates - Zimbabwe	41.04	39.77	16.32	18.24	89.5
**19-30 years child-bearing women**					
Insect samples	Protein g/100 g	Average portion size (g)	Protein in meal portion(g)	EAR (g/day)	% of EAR met
*G. belina* - SA	64.47	45.39	29.26	54.56	53.6
*G. belina* - Zimbabwe	64.26	45.39	29.17	54.56	53.5
*G. belina* - Zambia	62.99	45.39	28.56	54.56	52.4
*G. belina* - Botswana	62.49	45.39	28.36	54.56	52.0
*Gynanisa* caterpillar - SA	60.37	40.49	24.44	54.56	44.8
*Gynanisa* caterpillar - Zimbabwe	60.61	40.49	24.54	54.56	45.0
*Gynanisa* caterpillar - Botswana	59.61	40.49	24.14	54.56	44.2
Termite soldiers/workers - SA	69.75	15.27	10.65	54.56	19.5
Termite soldiers/workers - Zimbabwe	69.48	15.27	10.61	54.56	19.4
Termite alates - SA	42.27	39.77	16.81	54.56	30.8
Termite alates - Zimbabwe	41.04	39.77	16.32	54.56	29.9
**31-50 years child-bearing women**					
Insect samples	Protein g/100 g	Average portion size (g)	Protein in meal portion(g)	EAR (g/day)	% of EAR met
*G. belina* - SA	64.47	45.39	29.26	54.56	53.6
*G. belina* - Zimbabwe	64.26	45.39	29.17	54.56	53.5
*G. belina* - Zambia	62.99	45.39	28.59	54.56	52.4
*G. belina* - Botswana	62.49	45.39	28.36	54.56	52.0
*Gynanisa* caterpillar - SA	60.37	40.49	24.44	54.56	44.8
*Gynanisa* caterpillar - Zimbabwe	60.61	40.49	24.54	54.56	45.0
*Gynanisa* caterpillar - Botswana	59.61	40.49	24.14	54.56	44.2
Termite soldiers/workers - SA	69.75	15.27	10.65	54.56	19.5
Termite soldiers/workers - Zimbabwe	69.48	15.27	10.61	54.56	19.4
Termite alates - SA	42.27	39.77	16.81	54.56	30.8
Termite alates - Zimbabwe	41.04	39.77	16.32	54.56	29.9

**Table 3:** Essential amino acids of edible insects from different localities (g/100 g, dry mass basis)^1^

Treatments	Moisture	Histidine	Threonine	Lysine	Methionine
*G. belina* – SA	7.11^f^ ±0.26	0.67^b^ ±0.17	1.02^a^ ±0.33	1.02^a^ ±0.22	0.46^a^ ±0.09
*G. belina* – Botswana	6.43^d^ ±0.13	0.60^a^ ±0.48	1.44^b^ ±0.08	2.09^c^ ±0.07	0.70^a^ ±0.02
*G. belina* – Zambia	6.93^e^ ±0.10	0.67^b^ ±0.02	1.00^a^ ±0.13	1.19^a^ ±0.06	0.49^a^ ±0.08
*G. belina* – Zimbabwe	6.86^e^ ±0.15	0.51^a^ ±0.07	1.05^a^ ±0.02	1.23^a^ ±0.01	0.55^a^ ±0.01
*Gynanisa* caterpillar - SA	6.36^d^ ±0.08	0.55^a^ ±0.06	0.79^a^ ±0.07	0.80^a^ ±0.13	0.36^a^ ±0.10
*Gynanisa* caterpillar – Botswana	5.72^b^ ±0.18	0.44^a^ ±0.10	1.27^b^ ± 0.34	1.72^d^ ±0.06	0.44a ±0.17
*Gynanisa* caterpillar – Zimbabwe	6.10^c^ ±0.19	1.25^d^ ±0.01	2.64^d^ ±0.26	4.53^a^ ± 0.10	1.15^b^ ±0.17
termite soldiers/workers – SA	6.94^e^ ±0.15	0.96^c^ ±0.47	1.61^d^ ± 0.54	2.06^c^ ±0.08	1.17^b^ ±0.58
termite soldiers/workers – Zimbabwe	6.89^e^ ±0.20	1.22^d^ ±0.16	2.30^d^ ±0.11	2.77^b^ ±0.66	1.78^c^ ±0.06
Termite alates – SA	3.88^a^ ±0.94	1.07^d^ ±0.12	1.67^d^ ±0.20	2.28^d^ ± 0.42	1.91e ±0.09
Termite alates – Zimbabwe	7.07^f^ ±0.41	1.11^c^ ±0.02	1.52^c^ ± 0.18	2.14^b^ ±0.05	1.86^d^ ±0.04
p values	**0.001**	**0.001**	**0.001**	**0.001**	**0.001**
**Treatments**	**Valine**	**Isoleucine**	**Leucine**	**Phenylalanine**	**Sum of essential amino acids**
*G. belina*– SA	1.06^a^ ±0.21	0.78a ±0.14	1.16^a^ ±0.21	1.09^a^ ±0.27	7.26
*G. belina* – Botswana	1.62^b^ ±0.06	1.18^b^ ±0.01	1.75^b^ ± 0.04	1.43^b^ ± 0.11	10.81
*G. belina* – Zambia	1.09^a^ ±0.17	0.80^a^ ±0.11	1.21^a^ ± 0.16	1.10^a^ ±0.18	7.55
*G. belina* – Zimbabwe	1.12^a^ ±0.07	0.83^a^ ±0.02	1.24^a^ ± 0.05	1.03^a^ ± 0.07	7.56
*Gynanisa* caterpillar - SA	0.79^a^ ±0.00	0.63^a^ ±0.02	0.90^a^ ±0.01	0.83^a^ ±0.05	5.65
*Gynanisa* caterpillar – Botswana	1.17^b^ ±0.02	0.83^a^ ±0.08	1.29^a^ ±0.06	1.09^a^ ±0.01	8.25
*Gynanisa* caterpillar – Zimbabwe	3.30^d^ ±0.36	2.33^c^ ±0.14	3.50^d^ ±0.20	3.51^d^ ± 0.04	22.21
termite soldiers/workers – SA	2.20^c^ ±0.83	1.38^b^ ±0.50	2.46^b^ ±0.88	1.38^b^ ±0.72	13.22
termite soldiers/workers – Zimbabwe	3.47^d^ ±0.02	2.16^c^ ±0.03	3.87^d^ ±0.05	3.55^d^ ±0.04	21.12
Termite alates – SA	2.12^c^ ±0.22	1.56^b^ ±0.02	2.88^c^ ± 0.03	2.89^d^ ±0.25	16.38
Termite alates – Zimbabwe	2.01^c^ ±0.22	1.44^b^ ±0.13	2.65^b^ ±0.22	2.81^c^ ±0.13	15.54
p values	**0.001**	**0.001**	**0.001**	**0.001**	

1 Mean±SD, mean of six replicates; Means marked by different letters in the same column are significantly different (p<0.05).

**Table 4:** Mineral element content of edible insects from different countries (mg/kg, dry basis)^1^

Insect type	Moisture	Iron	Zinc
*G. belina* – SA	7.11^f^ ±0.26	97.0^g^ ±16.02	125.67^b^ ±15.50
*G. belina* - Botswana	6.43^d^ ±0.13	201.17^h^ ±53.84	129.0^c^ ±8.03
*G. belina* - Zambia	6.93^e^ ±0.10	170.83^i^ ±9.28	133.5^b^ ±16.73
*G. belina* -Zimbabwe	6.86^e^ ±0.15	290.66^j^ ±213.7	108.33^b^ ±41.3
*Gynanisa* caterpillar – SA	6.36^d^ ±0.08	367.83^d^ ±139.4	150.33^a^ ±10.02
*Gynanisa* caterpillar - Botswana	5.72^b^ ±0.18	230.16^e^ ±14.6	122.14^d^ ±8.72
*Gynanisa* caterpillar - Zimbabwe	6.10^c^ ±0.19	241.33^e^ ±8.42	148.83^a^ ±11.55
Termite soldiers/workers – SA	6.94^e^ ±0.15	545.0^c^ ±27.97	142.66^a^ ±1.54
Termite soldiers/workers - Zimbabwe	6.89^e^ ±0.20	629.5^a^ ±26.55	131.16^c^ ±3.62
Termite alates - SA	3.88^a^ ±0.94	307.67^g^ ±160.42	109.66^e^ ±6.82
Termite alates - Zimbabwe	7.07^f^ ±0.41	177.17^i^ ±38.60	109.33^e^ ±7.96
p value	**0.01**	**0.001**	**0.001**

1 Mean±SD, mean of six replicates; Different letters in columns show significant difference (p<0.05).

**Table 5:** Percentage of the Estimated Average Requirement met for Iron and Zinc for different age groups from the consumption of usual portions of dried edible insects from different countries

4-8 years old children					
Insect samples	Iron in insect (100/kg)	Usual portion size (kg)	Iron in meal portion (mg)	EAR (mg/day)	% of EAR met
*G. belina* - SA	97.0	0.045	4.40	4.1	107.4
*G. belina* - Zimbabwe	290.7	0.045	13.19	4.1	321.8
*G. belina* – Zambia	170.8	0.045	7.75	4.1	189.1
*G. belina* - Botswana	201.2	0.045	9.13	4.1	222.7
*Gynanisa* caterpillar - SA	385.8	0.040	15.62	4.1	381.0
*Gynanisa* caterpillar - Zimbabwe	241.3	0.040	9.77	4.1	238.3
*Gynanisa* caterpillar - Bots	230.2	0.040	9.32	4.1	227.3
Termite soldiers/workers - SA	545.0	0.015	8.32	4.1	203.0
Termite soldiers/workers - Zimbabwe	629.5	0.015	9.61	4.1	234.5
Termite alates - SA	307.7	0.040	12.24	4.1	298.4
Termite alates - Zimbabwe	177.2	0.040	7.05	4.1	171.9
Insect samples	Zinc in insect (mg/kg)	Usual portion size (kg)	Zinc in meal portion (mg)	EAR (mg/day)	% of EAR met
*G. belina* - SA	125.67	0.045	5.70	4	142.6
*G. belina* - Zimbabwe	108.34	0.045	4.92	4	122.9
*G. belina* – Zambia	133.50	0.045	6.06	4	151.5
*G. belina* - Botswana	129.00	0.045	5.86	4	146.4
*Gynanisa* caterpillar - SA	150.34	0.040	6.09	4	152.2
*Gynanisa* caterpillar - Zimbabwe	148.83	0.040	6.03	4	150.6
*Gynanisa* caterpillar - Bots	122.17	0.040	4.59	4	123.7
Termite soldiers/workers - SA	142.67	0.015	2.18	4	54.5
Termite soldiers/workers - Zimbabwe	131.84	0.015	2.01	4	50.3
Termite alates - SA	109.67	0.040	4.36	4	109.0
Termite alates - Zimbabwe	109.34	0.040	4.35	4	108.7
**19-30 years child-bearing women**					
Insects samples	Iron in insect (mg/kg)	Usual portion size (kg)	Iron in meal portion (mg)	EAR (mg/day)	% of EAR met
*G. belina* - SA	97.0	0.045	4.40	22	20.0
*G. belina* - Zimbabwe	290.7	0.045	13.19	22	60.0
*G. belina*– Zambia	170.8	0.045	7.75	22	35.3
*G. belina* - Botswana	201.2	0.045	9.13	22	41.5
*Gynanisa* caterpillar - SA	385.8	0.040	15.62	22	71.0
*Gynanisa* caterpillar - Zimbabwe	241.3	0.040	9.77	22	44.4
*Gynanisa* caterpillar - Bots	230.1	0.040	9.32	22	42.4
Termite soldiers/workers - SA	545.0	0.015	8.32	22	37.8
Termite soldiers/workers - Zimbabwe	629.5	0.015	9.61	22	43.7
Termite alates - SA	307.7	0.040	12.24	22	55.6
Termite alates - Zimbabwe	177.2	0.040	7.05	22	32.0
Insect samples	Zinc in insect (mg/kg)	Usual portion size (kg)	Zinc in meal portion (mg)	EAR (mg/day)	% of EAR met
*G. belina* - SA	125.7	0.045	5.70	9.5	60.0
*G. belina* - Zimbabwe	108.3	0.045	4.92	9.5	51.8
*G. belina* – Zambia	133.5	0.045	6.06	9.5	63.8
*G. belina* - Botswana	129.0	0.045	5.86	9.5	61.6
*Gynanisa*caterpillar - SA	150.3	0.040	6.09	9.5	64.1
*Gynanisa* caterpillar - Zimbabwe	148.8	0.040	6.03	9.5	63.4
*Gynanisa* caterpillar - Bots	122.2	0.040	4.95	9.5	52.1
Termite soldiers/workers - SA	142.7	0.015	2.18	9.5	22.9
Termite soldiers/workers - Zimbabwe	131.8	0.015	2.01	9.5	21.2
Termite alates - SA	109.7	0.040	4.36	9.5	45.9
Termite alates - Zimbabwe	109.3	0.040	4.35	9.5	45.8
**31-50 years child-bearing women**					
Insect samples	Iron in insect (mg/kg)	Usual portion size (kg)	Iron in meal portion (mg)	EAR (mg/day)	% of EAR met
*G. belina* - SA	97.0	0.045	4.40	22	20.1
*G. belina* - Zimbabwe	290.7	0.045	13.19	22	60.0
G*. belina* – Zambia	170.8	0.045	7.75	22	35.3
*G. belina* - Botswana	201.2	0.045	9.13	22	41.5
*Gynanisa* caterpillar - SA	385.8	0.040	15.62	22	71.0
Gynanisa caterpillar - Zimbabwe	241.3	0.040	9.77	22	44.4
Gynanisa caterpillar - Bots	230.2	0.040	9.32	22	42.4
Termite soldiers/workers - SA	545.0	0.015	8.32	22	37.8
Termite soldiers/workers - Zimbabwe	629.5	0.015	9.61	22	43.7
Termite alates - SA	307.7	0.040	12.24	22	55.6
Termite alates - Zimbabwe	177.2	0.040	7.05	22	32.0
Insect samples	Zinc in insect (mg/kg)	Usual portion size (kg)	Zinc in meal in portion (mg)	EAR (mg/day)	% of EAR met
*G. belina* - SA	125.7	0.045	5.70	9.5	60.0
*G. belina* - Zimbabwe	108.3	0.045	4.92	9.5	51.8
*G. belina* – Zambia	133.5	0.045	6.06	9.5	63.8
*G. belina* - Botswana	129.0	0.045	5.86	9.5	61.6
*Gynanisa* caterpillar - SA	150.3	0.040	6.09	9.5	64.1
*Gynanisa* caterpillar - Zimbabwe	148.8	0.040	6.03	9.5	63.4
Gynanisa caterpillar - Bots	122.1	0.040	4.95	9.5	52.1
Termite soldiers/workers - SA	142.7	0.015	2.18	9.5	22.9
Termite soldiers/workers - Zimbabwe	131.8	0.015	2.01	9.5	21.2
Termite alates - SA	109.7	0.040	4.36	9.5	45.9
Termite alates - Zimbabwe	109.3	0.040	4.35	9.5	45.8

**Table 6:** Proximate composition of *G. belina* samples cooked by different cooking methods ^1^

Treatments	Moisture	Protein	Fat	Ash	Energy (MJ/kg)
Dried *G. belina*(control)	6.47^a^ ±0.26	65.02^b^ ±0.38	14.10^c^ ±0.68	9.91^a^ ±0.93	18.89^c^ ±2.22
Boiled *G. belina*	12.72^c^ ±0.69	66.85^d^ ±0.56	13.06^b^ ±0.30	11.81^c^ ±0.36	18.09^b^ ±0.07
Boiled, salted *G. belina*	16.59^a^ ±0.34	66.14^c^ ±0.37	13.44^a^ ±0.53	11.87^c^ ±0.73	18.08^b^ ±0.08
Fried *G. belina*	11.69^b^ ±0.34	60.74^a^ ±0.64	20.57^d^ ±1.36	11.5^b^ ±0.32	19.77^a^ ±1.46
p value	**0.001**	**0.00**	**0.001**	**0.001**	**0.001**

1 Mean±SD, mean of nine replicates; means marked by different letters in the same column are significantly different (p<0.05). Nutrient values are on a dry matter basis (g/100 g).

**Table 7:** Effect of cooking method on the essential amino acid content of *G. belina* (g/100 g, dry mass basis)

Treatments	Dried *G. belina*(control)	Boiled *G. belina*	Boiled, salted *G. belina*	Fried *G. belina*	p value
Moisture	6.47^a^ ±0.26	12.72^c^ ±0.69	16.59^a^ ±0.34	11.69^b^ ±0.34	**0.001**
Histidine	1.98^a^ ±0.3	1.50^c^ ±0.32	1.26^b^ ±0.13	2.24^d^ ± 0.34	**0.006**
Threonine	3.0^c^ ±0.11	2.63^a^ ± 0.1	2.71^b^ ±0.12	2.53^d^ ±0.03	**0.005**
Lysine	5.05^d^ ±0.99	4.70^c^ ±0.38	4.31^b^ ±0.85	2.94^a^ ±0.35	**0.025**
Methionine	1.28^c^ ±0.28	1.65^b^ ± 0.13	1.42^a^ ±0.19	1.46^a^ ±0.05	**0.004**
Valine	3.3^b^ ±0.11	3.07^c^ ± 0.08	2.89^a^ ±0.06	3.02^a^ ±0.03	**0.001**
Isoleucine	2.38±0.23	2.25 ±0.15	2.08 ±0.10	1.96 ±0.02	0.091
Leucine	3.65 ±0.37	3.44 ±0.24	3.17±0.19	3.1±0.03	0.204
Phenylalanine	3.69± 0.48	3.77± 0.19	3.38 ±0.49	3.56±0.15	0.824

1 Mean±SD, mean of nine replicates; Means marked by different letters in the same column are significantly different (p<0.05)

**Table 8:** Effect of cooking method on the iron and zinc content of *G. belina* (mg/kg, dry basis)

Cooking method	Moisture	Iron	Zinc
Dried *G. belina* (control)	6.47^a^±0.26	95.33^a^ ±10.99	108.2^a^±10.5
Boiled *G. belina*	12.72^c^±0.69	149.77^b^±18.77	135.3^c^±14.23
Boiled, salted *G. belina*	16.59^a^±0.34	178.8^c^±16.11	134.4^b^±6.47
Fried *G. belina*	11.69^b^±0.34	191.44^d^±3.08	133.53^b^±10.39
p value	**0.001**	**0.001**	**0.001**

1 Mean±SD, mean of nine replicates. Means marked by different letters in the same column are significantly different (p<0.05)
